# Assessing the predictive value of clinical factors to pathological complete response for locally advanced rectal cancer: An analysis of 124 patients

**DOI:** 10.3389/fonc.2023.1125470

**Published:** 2023-03-31

**Authors:** Chaoxi Zhou, Kanghua Wang, Xiaoxiao Zhang, Yuting Xiao, Congrong Yang, Jun Wang, Fuyin Qu, Xuan Wang, Ming Liu, Chao Gao, Linlin Xiao, Fengpeng Wu

**Affiliations:** ^1^ Department of General Surgery, Fourth Hospital of Hebei Medical University, Shijiazhuang, Hebei, China; ^2^ Department of Medical Oncology, Affiliated Hospital Of Hebei University, Baoding, China; ^3^ Department of Radiotherapy, Fourth Hospital of Hebei Medical University, Shijiazhuang, China; ^4^ Department of Radiation Oncology, Hebei Cancer Hospital Chinese Academy of Medical Sciences, Langfang, China

**Keywords:** locally advanced rectal cancer, neoadjuvant chemoradiotherapy, extramural vascular invasion, carcino-embryonic antigen (CEA), pathological complete response (PCR)

## Abstract

**Purpose:**

To investigate the clinical factors affecting pathological complete response (pCR) after neoadjuvant chemoradiotherapy (nCRT) in locally advanced rectal cancer (LARC).

**Methods:**

Clinical data of 124 LARC patients treated with nCRT and surgery in the fourth Hospital of Hebei Medical University from 2014 to 2019 were retrospectively analyzed. In this study, univariate analysis and logistic dichotomous multivariate regression analysis were used to study the clinical factors affecting pCR, and the receiver operator characteristic curve (ROC) analysis was used to further verify the accuracy of partial indexes in predicting pCR.

**Results:**

Of the 124 enrolled patients, 19 patients (15.32%) achieved pCR. Univariate analysis showed that the number of cycles of consolidation chemotherapy, serum carcino-embryonic antigen (CEA) level before treatment, MRI longitudinal length of tumor, and extramural vascular invasion (EMVI) were statistically correlated with pCR. ROC analysis of the longitudinal length of tumor measured by MRI showed that the area under the curve (AUC) value, sensitivity and specificity were 0.735, 89.47% and 48.57% respectively, and the optimal cut-off value was 5.5cm. The ROC analysis showed that the AUC value, sensitivity and specificity of pCR prediction using CEA were 0.741, 63.16% and 90.48%, respectively, and the optimal cut-off value was 3.1ng/ml. Multivariate results showed that the number of cycles of consolidation chemotherapy, serum CEA level before treatment, and EMVI were independent predictors of pCR.

**Conclusion:**

The number of cycles of consolidation chemotherapy, serum CEA level before treatment, and EMVI may be important determinants of LARC patients to reach pCR after nCRT.

## Introduction

Neoadjuvant chemoradiotherapy (nCRT) had the advantages of reducing local recurrence rate (LRR) and improving sphincter retention rate (1, 2). Therefore, the National Comprehensive Cancer Network (NCCN) guidelines recommended nCRT for patients with locally advanced rectal cancer (LARC) (3, 4). LARC patients have distinct individual differences in response to nCRT. About 54%-75% of patients could achieve tumor staging reduction after nCRT, and only 9%-25% could achieve pathological complete response (pCR) (5–7). Patients who achieved pCR had better prognosis, lower LRR, and lower distant metastasis rate, with a 5-year overall survival (OS) of 87.6% and a 5-year LRR of only 2.8% (8–10). At present, some studies suggested that when patients achieve clinical complete response (cCR), a “watch and wait”, nonoperative (chemotherapy and/or RT) management approach may be considered to replace the total mesorectal excision (TME) in centers with experienced multidisciplinary teams (11, 12). By this management, surgery-related complications including intestinal function, urinary tract and sexual dysfunction could be avoided, thereby improving the quality of life of patients (13). Therefore, patients with pCR may be more suitable for this treatment strategy. However, patients who achieve cCR do not necessarily achieve pCR after surgery. Studies have shown that about 25% of patients with cCR are confirmed as pCR (14). At present, pCR is mainly confirmed by histopathological diagnosis of postoperative specimens. There are no accurate, reliable and non-invasive clinical predictors for pCR. Therefore, finding clinically relevant factors that predict pCR in LARC patients after nCRT may avoid unnecessary radical surgery, which has a significant meaning for individualized treatment of patients. This study aims to explore the clinical factors affecting the pCR of LARC patients after nCRT, so as to guide patients to optimize the treatment plan and predict the prognosis of patients.

## Materials and methods

### Patients

LARC patients who completed nCRT combined with TME surgery in the Fourth Hospital of Hebei Medical University from January 2014 to December 2019, were included in this retrospective case control study according. Patients were grouped according to tumor regression grading after nCRT.

The inclusion criteria were as follows: (1) Histopathology was confirmed rectal adenocarcinoma before neoadjuvant therapy; (2) T3-4, N0/N+, and M0 were diagnosed by imaging examination (chest CT, abdominal and pelvic MRI, PET-CT) at initial diagnosis; (3) Neoadjuvant therapy and TME surgery were completed before entering this study; (4) The mode of neoadjuvant therapy was long- course concurrent chemoradiotherapy recommended by NCCN guidelines.

The exclusion criteria were as follows: (1) Patients have other malignancies besides rectal cancer; (2) Distant metastases were found before surgery; (3) The neoadjuvant therapy was chemotherapy alone, radiotherapy alone, short-course radiotherapy (SCRT) or induction chemotherapy before radiotherapy; (4) Patients have incomplete clinical data.

All patients were treated with long-course preoperative RT by intensity-modulated radiation therapy (IMRT) using 6 MV photons. The median dose of radiotherapy was 50.4Gy (45-70Gy), including 114 cases with ≤50.4Gy and 10 cases with >50.4Gy, and the single dose was 1.8-2.0Gy. The target volume delineation and field setup were completed with reference to the ICRU Report 83 and the academic writings of Lee et al. (15). The chemotherapy regimens concurrently with irradiation were as follows: Capecitabine (82 cases), 5-FU+ calcium Leucovorin (3 cases), FOLFOX (9 cases), and XELOX (30 cases).

The collection of clinical data was approved by the ethics committee of the fourth hospital of Hebei Medical University. The data are anonymous, and the requirement for informed consent was therefore waived.

### Statistical analysis

Statistical analysis was performed using SPSS software 22.0 (SPSS, Inc., Chicago, IL, USA). Chi-square test or Fisher exact test was used for univariate analysis. Logistic binary regression analysis (forward stepwise) was used for multivariate analysis to investigate the clinical factors affecting pCR, and the Hosmer-Lemeshow test was used to evaluate the goodness of fit of logistic regression model. In addition, the receiver operator characteristic curve (ROC) was used to calculate the area under the curve (AUC) to test some statistically significant variable values. In this study, P < 0.05 was considered statistically significant.

## Results

### Characteristics of patients

From January 2014 to December 2019, 203 LARC patients were found at the Fourth Hospital of Hebei Medical, of which 124 patients met the inclusion criteria of this study. The median patient age at the time of LARC diagnosis was 58 years old (30-87), including 95 males and 29 females. There were 87 patients with Dixon surgery, 34 patients with Miles surgery, and 3 patients with Hartman surgery. The anus preservation rate was 72.58%. In terms of the efficacy evaluation of nCRT, according to the tumor regression grading (AJCC 8th) standard (16), pathology experts identified 19 of 124 cases with tumor regression grading (TRG) 0, 13 with TRG 1, 78 with TRG 2, and 14 with TRG 3. In our study, patients with TRG 0-1 status were defined as good regression (GR). The main clinical characteristics of the patients were listed in **Table 1**.

**Table 1:** Characteristics of patients with or without pCR.

Characteristics	Patients Number	pCR	Non-pCR	*P value*
Gender				0.252
Male	95	17	78	
Female	29	2	27	
Age				0.821
30-49	33	4	29	
50-69	75	13	62	
≥70	16	2	14	
Concurrent chemotherapy regimens				0.600
Single-agent fluorouracil	85	14	71	
Oxaliplatin+platinum	39	5	34	
Radiation dose (Gy)				0.630
≤50.4	114	18	96	
>50.4	10	1	9	
Time between nCRT and surgery (week)				0.490
6≤X<8	10	0	10	
8≤X<10	36	5	31	
≥10	78	14	64	
Cycles of consolidation chemotherapy				**0.035**
0	54	4	50	
1-2	54	9	42	
>2	16	6	13	
T staging				0.273
T3	91	12	79	
T4	33	7	26	
N staging				
N0	5	0	5	0.247
N1	21	1	20	
N2	98	18	80	
Distance between tumor and anal border (cm)				0.538
<5	47	7	40	
5≤X<10	68	12	56	
10≤X<15	9	0	9	
Proportion of tumor in enteric cavity				0.097
<1/2	33	8	25	
≥1/2	91	11	80	
EMVI				**0.014**
No	84	18	66	
Yes	40	1	39	
CEA level (ng/mL)				**0.030**
<5	59	15	44	
5≤X<10	24	1	23	
10≤X<20	18	2	16	
≥20	23	1	22	
CA199 level (U/mL)				0.568
<30	100	14	86	
30≤X<60	11	2	9	
≥60	13	3	10	
NLR				0.399
<3	93	14	79	
3≤X<5	25	3	22	
≥5	6	2	4	
PLR				0.477
≤150	68	9	59	
>150	56	10	46	
Length (cm)				**0.027**
<5	50	12	38	
≥5	74	7	67	
The largest thickness (cm)				0.525
<1	10	1	9	
1≤X<2	81	11	70	
≥2	33	7	26	
Pathological type				0.716
mucinous adenocarcinoma	9	1	8	
non-mucinous adenocarcinoma	115	18	97	

pCR, pathological complete response; nCRT, neoadjuvant chemoradiotherapy; EMVI, extramural venous invasion; CEA, carcino-embryonic antigen; NLR, neutrophil to lymphocyte ratio; PLR, platelet-to-lymphocyte ratio. Bolded value means P value < 0.05.

### Univariate and multivariate analysis

Univariate analysis demonstrated that the number of cycles of consolidation chemotherapy (P=0.035), CEA level before treatment (P=0.030), longitudinal length of the tumor on MRI (P=0.027), and extramural vascular invasion (EMVI) or not (P=0.014) were significantly associated with pCR (**Table 1**).

The significance of the Hosmer-Lemeshow goodness of fit was 0.327, indicating that the model had a good degree of fit (P>0.05). After all factors listed in **Table 1** were brought into the logistic regression model as independent variables, we found that the cycle number of consolidation chemotherapy ≥1 (P=0.042), serum CEA before treatment <5ng/mL (P=0.005) and EMVI negative (P=0.045) were independent predictors of pCR and were significantly associated with higher pCR rate in LARC patients after nCRT (**Table 2**), and the longitudinal length of the tumor was not found to have independent predictive value, although this factor was found to have significant correlation with pCR in univariate analysis.

**Table 2:** Logistic multivariate analysis of pCR in LARC patients after nCRT.

Characteristics	OR (95% CI)	P value
Cycle number of consolidation chemotherapy	2.362 (1.031-5.41)	0.042
CEA level(ng/mL)	0.388 (0.199-0.754)	0.005
EMVI or not	0.115 (0.014-0.952)	0.045

pCR, pathological complete response; LARC, locally advanced rectal cancer; nCRT, neoadjuvant chemoradiotherapy; CEA, carcino-embryonic antigen; EMVI, extramural venous invasion.

#### ROC analysis

**Figure 1:**
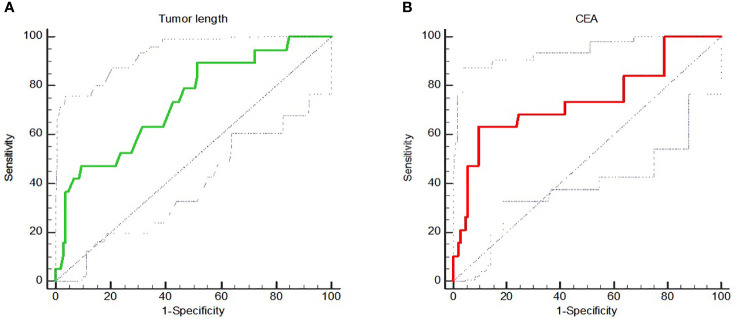
**(A)** showed the ROC analysis of correlation between longitudinal length of tumor measured by MRI and pCR; **(B)** showed the ROC analysis of correlation between CEA level and pCR.

ROC analysis of the longitudinal length of tumor measured by MRI showed that the AUC value, sensitivity and specificity were 0.735, 89.47% and 48.57% respectively, and the optimal cut-off value was 5.5cm. The ROC analysis of the correlation between CEA level before treatment and pCR showed that the AUC value, sensitivity and specificity of pCR prediction using CEA were 0.741, 63.16% and 90.48%, respectively, and the optimal cut-off value was 3.1ng/mL (**Figure 1**).

## Discussion

Colorectal cancer is the third most common malignancy and the second leading cause of cancer death worldwide (17). LARC patients with pCR have higher local control rate, lower distant metastasis rate, and better survival (10). Whereas, there was still no reliable clinical predictor of pCR. This study enrolled 124 LARC patients, which demonstrated that the number of cycles of consolidation chemotherapy, serum CEA level before treatment, and EMVI may be important determinants of LARC patients to reach pCR after nCRT.

CEA is a glycoprotein secreted by colorectal cancer tissues and a common tumor marker of colorectal cancer. It is of great value in clinical screening, disease progression monitoring and prognosis prediction of colorectal cancer patients. At present, some studies have found that pre-treatment CEA level is still of great significance in predicting pCR (18–22). Cheong et al. (18) retrospectively studied 145 LARC patients who received nCRT and found that 92.6% patients with pCR showed pre-treatment CRT CEA levels <5 ng/mL (P<0.001). Pre-treatment CRT CEA levels were important risk factors for pCR (OR=18.71; 95%CI:4.62–129.51, P<0.001), respectively. Li et al. (19) found that the pre-treatment CEA level of patients in the pCR group was significantly lower than that of patients in the non-pCR group (3.82 ± 4.08 vs. 25.33 ± 49.41). It was a significant predictor of pCR, with AUC of 0.785 and optimal cut-off value of 3.35 ng/mL. These results indicated that the level of pre-treatment CEA may be a reasonable biomarker for predicting the pathological response of rectal cancer. However, the optimal cut-off value of pre-treatment CEA level to predict pCR is still inconsistent (19–22). In addition, contrary to the above studies, some studies did not find a correlation between CEA level and pCR (23, 24). The reasons for these divergences may be the differences in the enrolled population and the relatively small sample size of the retrospective studies. Further multi-institutional, prospective studies with a large sample size or meta-analysis studies were needed to confirm these findings.

It is well known that LARC patients who achieve pCR have a good prognosis, but only a small proportion of patients could achieve pCR after nCRT. In order to improve the tumor downstaging rate and achieve higher pCR rate, some studies have proposed total neoadjuvant therapy (TNT), which means the addition of consolidation chemotherapy after nCRT. Earlier study by Garcia-Aguilar et al. (25) proposed that nCRT followed by consolidation chemotherapy could improve pCR rate in a multi-center phase II clinical trial. This study demonstrated that the pCR rate of patients with nCRT and consolidation chemotherapy was higher than that of patients with nCRT alone (P=0.0036). Compared with the nCRT alone group, nCRT followed by 6 cycles of consolidation chemotherapy could bring a significantly higher survival advantage (OR=3.49, 95%CI 1.39-8.75; P=0.011). Liang et al. (26) found that patients in the nCRT followed by consolidation chemotherapy group had significantly higher “pCR rate + near-pCR rate” (32.8% vs. 16.25%; P=0.015), the univariate analysis and multivariate analysis found that consolidation chemotherapy was the independent predictor to achieve high “pCR rate and close to pCR rate”. In addition, the research showed that the consolidation chemotherapy was safe and feasible. There were no difference between the two groups in grade 3 to 4 toxic effects (nausea, vomiting, lower white blood cell count and anemia, etc.). Zhai et al. (27) found that the pCR rate of patients in the nCRT alone group was only 12.8%, while it was 32.7% in the nCRT followed by 3 cycles of XELOX consolidation chemotherapy. Although consolidation chemotherapy improved the pCR rate of patients, the rate of grade 3-4 adverse reactions did not increase. The univariate analysis showed that consolidation chemotherapy was an independent predictor of pCR.

Although some studies showed that the TNT regimen could improve pCR rate, some studies still presented different opinions. A phase II clinical trial of KCSG CO 14-03 by Kim et al. (28) showed that the pCR rate of patients in the nCRT group alone was 5.8%, while the pCR rate of patients in the nCRT followed by 2 cycles of XELOX consolidation chemotherapy group was 13.6%. Although the pCR rate of patients in the consolidation chemotherapy group was slightly higher than that of patients in the non-consolidation chemotherapy group, the difference was not statistically significant. Moore et al. analyzed 49 LARC patients and showed that the pCR rate of patients in the nCRT followed by consolidation chemotherapy group was 16%, while that in the nCRT alone group was as high as 25% (29). Therefore, the researcher thought that consolidation chemotherapy was not helpful to improve the pCR rate of patients.

So, could consolidation chemotherapy improve the pCR rate in patients? There were two meta-analysis studies. The study of Riesco-Martinez et al. showed that the pCR rate of patients in the consolidation chemotherapy group was significantly higher than that in the non-consolidation chemotherapy group (22.9% vs 13.2%, P<0.001) (30). In addition, no significant increase in grade 3-4 toxicity was observed in consolidation chemotherapy regimens. The study of Petrelli et al. showed that the addition of TNT treatment with induction chemotherapy and/or consolidation chemotherapy could improve the pCR rate of patients, and the toxicity of TNT regimen was comparable to that of standard treatment regimen (31). These studies suggest that consolidation chemotherapy may be helpful and safe to improve the pCR rate of patients.

EMVI refers to the presence of tumor cells in the blood vessels outside the muscularis propria (32). The characteristics of EMVI on MRI are that tumor signals exist in the vascular structure, blood vessels dilate or tumor infiltrates beyond the vascular wall and destroys the vascular boundary (32, 33). EMVI was associated with a higher risk of distant metastasis and poor prognosis (34, 35). In addition, EMVI was an independent predictor of higher recurrence risk in LARC patients after nCRT (36). At present, there were few studies on EMVI in predicting responsiveness to nCRT in LARC patients with different conclusions. A study of 649 LARC patients undergoing nCRT by the European Colorectal Cancer Association showed that the pCR rate and partial response rate of EMVI positive patients were lower than those of EMVI negative patients (7.5% vs 86.6%, 6.9% vs 83.3%), but the difference was not statistically significant (37). Hammarstrom et al. (38) showed that among patients in the short-course radiotherapy group, the cCR rate of EMVI negative patients was significantly higher than that of EMVI positive patients (11% vs 0%, P=0.017). While in the nCRT group and short-course radiotherapy followed by consolidation chemotherapy group, the cCR rate in EMVI negative patients was comparable to that in EMVI positive patients (16% vs. 18%, 29% vs. 23%). So, researchers suggested that EMVI positive may only be a predictor of poor sensitivity to radiotherapy. Sun et al. (39) analyzed the value of EMVI on the response to nCRT in patients with stage T3. The study showed that the good response rate of EMVI negative patients was about twice that of EMVI positive patients. Multivariate analysis showed that EMVI negative was an independent predictor of good response to rectal cancer. This study showed that the pCR rate of EMVI negative patients was significantly higher than that of EMVI positive patients (21.4% vs 2.5%, P=0.014). Multivariate analysis showed that EMVI negative was an independent predictor of pCR. In conclusion, studies using EMVI to predict the nCRT sensitivity are rare and controversial in LARC patients, further studies are needed to confirm the accuracy of this finding.

Tumor diameter or longitudinal length, which reflect tumor size, may be another clinical factor affecting pCR achievement in LARC patients. Garland et al. (23) studied 297 LARC patients who received nCRT and found that patients with smaller tumors were more likely to achieve pCR (5.0 ± 2.0cm vs. 6.0 ± 2.0, P = 0.008). Multivariate analysis showed that tumor size under endoscopy was an independent predictor of pCR. However, when the analysis was stratified by tumor size of <3.5cm, 3.5-7cm and >7 cm, the results showed that there was no correlation between tumor size and pCR (P=0.094). Park et al. (40) studied 249 LARC patients, and the univariate analysis showed that the proportion of pCR rate in patients with tumor size ≤4cm was significantly higher (37.61% vs. 18.40%, P=0.001), but multivariate analysis showed that tumor size was not a predictor of pCR. In the study of Lee et al. (41), the cut-off value of tumor size was set as 5cm, which was consistent with Park et al. (40), and only the results of univariate analysis showed that tumor size was correlated with pCR. Univariate analysis by Russo et al. (42) showed that patients with smaller tumors were more likely to achieve pCR, but the study did not provide cut-off value for grouping and conduct multivariate analysis to further confirm the accuracy of this conclusion. In this study, univariate analysis showed that patients with longitudinal tumor length <5cm were more likely to achieve pCR than those with longitudinal tumor length ≥5cm (24.00% vs. 9.46%, P=0.027). ROC analysis showed that the AUC value, sensitivity and specificity were 0.735, 89.47% and 48.57% respectively, and the optimal cut-off value was 5.5cm. However, multivariate analysis did not show statistical significance. To sum up, the conclusions of various studies are different, and it is not certain whether tumor size is the factor affecting the pCR achievement of LARC patients. In addition, the cut-off value of tumor size classification may also be an important factor affecting the results, which should be fully paid attention to in future studies.

As we all know, radiotherapy plays a major role in the neoadjuvant treatment of LARC patients. Although the recommended irradiation dose for LARC patients is 45Gy/25F to PTV and 50.4Gy/28F to CTV-H according to NCCN guidelines, there is still controversy about whether further increase of radiotherapy dose can improve the tumor regression. Appelt et al. performed 60Gy external irradiation sequential 5Gy brachytherapy boost on 51 patients with T2-3/N0-1, and found that 40 cases (78.4%) obtained cCR and entered “Watch & Wait” (43). Another prospective study compared the tumor regression of LARC patients receiving 50.4Gy and 60Gy, and found that increased dose did not significantly improve the pCR of patients, but the downstaging and shrinking of primary tumors were more significant in high-dose group of T3 patients (p=0.049), although there was no significant difference in the pathological reaction of lymph nodes between the two groups (44). In our study, the dose of enrolled patients ranged from 45Gy to 70Gy, and the results showed that only 1 out of 10 patients with a dose greater than 50.4Gy obtained pCR, and the tumor regression status of the patients might not benefit from high dose irradiation.

In this retrospective study, considering the reality of low pCR in patients receiving SCRT, we only analyzed patients with long-course nCRT. In addition, since some patients did not undergo genetic testing, the status of RAS, BRAF and MMR was not included, and only the general characteristics and those significant factors mentioned in other studies were analyzed, which might lead to some potential confounders to interfere with our results. We will pay attention to the above limitations and avoid them as much as possible in the design of future prospective studies.

In conclusion, this study demonstrated that the number of cycles of consolidation chemotherapy, serum CEA level before treatment, and EMVI may be important determinants of LARC patients to reach pCR after nCRT. Whereas, this is a retrospective study with small sample. Further multi-institutional, prospective studies with a large sample size or meta-analysis studies are needed to confirm these findings.

## Data availability statement

The datasets presented in this study can be found in online repositories. The names of the repository/repositories and accession number(s) can be found in the article/**Supplementary Material**.

## Ethics statement

The studies involving human participants were reviewed and approved by the fourth hospital of Hebei Medical University. Written informed consent for participation was not required for this study in accordance with the national legislation and the institutional requirements.

## Author contributions

Conceived and designed the study: FW and LX. Performed the study and analyzed the data: KW, XZ, CY, LX, FQ, JW, XW, CG, and ML. Wrote the paper: KW, LX, CZ, FW, and YX. Supervised the entire study and review the final paper: FW, CZ, LX, and KW. All authors contributed to the article and approved the submitted version.

## References

[1] Benson AB 3rd Venook AP Bekaii-Saab T Chan E Chen YJ Cooper HS. Rectal cancer, version 2.2015. J Natl Compr Canc Netw (2015) 13(6):719–28. doi: 10.6004/jnccn.2015.0087 26085388

[2] Sauer R Liersch T Merkel S Fietkau R Hohenberger W Hess C. Preoperative versus postoperative chemoradiotherapy for locally advanced rectal cancer: Results of the German CAO/ARO/AIO-94 randomized phase III trial after a median follow-up of 11 years. J Clin Oncol (2012) 30(16):1926–33. doi: 10.1200/JCO.2011.40.1836 22529255

[3] Yang KL Yang SH Liang WY Kuo YJ Lin JK Lin TC. Carcinoembryonic antigen (CEA) level, CEA ratio, and treatment outcome of rectal cancer patients receiving pre-operative chemoradiation and surgery. Radiat Oncol (2013) 8:43. doi: 10.1186/1748-717X-8-43 23452434 PMC3599903

[4] Tan Y Fu D Li D Kong X Jiang K Chen L. Predictors and risk factors of pathologic complete response following neoadjuvant chemoradiotherapy for rectal cancer: A population-based analysis. Front Oncol (2019) 9:497. doi: 10.3389/fonc.2019.00497 31263674 PMC6585388

[5] Fernandez-Martos C Aparicio J Bosch C Torregrosa M Campos JM Garcera S. Preoperative uracil, teg -afur, and concomitant radiotherapy in operable rectal cancer: A phase II multicenter study with 3 years' follow-up. J Clin Oncol (2004) 22(15):3016–22. doi: 10.1200/JCO.2004.11.124 15210740

[6] Kim YH Kim DY Kim TH Jung KH Chang HJ Jeong SY. Usefulness of magnetic resonance volumetric evaluation in predicting response to preoperative concurrent chemoradiotherapy in patients with resectable rectal cancer. Int J Radiat Oncol Biol Phys (2005) 62(3):761–8. doi: 10.1016/j.ijrobp.2004.11.005 15936557

[7] Edge SB Compton CC. The American joint committee on cancer: the 7th edition of the AJCC cancer staging manual and the future of TNM. Ann Surg Oncol (2010) 17(6):1471–4. doi: 10.1245/s10434-010-0985-4 20180029

[8] Maas M Nelemans PJ Valentini V Das P Rödel C Kuo LJ. Long-term outcome in patients with a pathological complete response after chemoradiation for rectal cancer: A pooled analysis of individual patient data. Lancet Oncol (2010) 11(9):835–44. doi: 10.1016/S1470-2045(10)70172-8 20692872

[9] Rödel C Martus P Papadoupolos T Füzesi L Klimpfinger M Fietkau R. Prognostic significance of tumor regression after preoperative chemoradiotherapy for rectal cancer. J Clin Oncol (2005) 23(34):8688–96. doi: 10.1200/JCO.2005.02.1329 16246976

[10] Martin ST Heneghan HM Winter DC. Systematic review and meta-analysis of outcomes following pathological complete response to neoadjuvant chemoradiotherapy for rectal cancer. Br J Surg (2012) 99(7):918–28. doi: 10.1002/bjs.8702 22362002

[11] Habr-Gama A Perez RO Nadalin W Sabbaga J Ribeiro U Jr Silva e Sousa AH Jr. Operative versus nonoperative treatment for stage 0 distal rectal cancer following chemoradiation therapy: Long-term results. Ann Surg (2004) 240(4):711–7. doi: 10.1097/01.sla.0000141194.27992.32 PMC1356472 15383798

[12] Yang TJ Goodman KA. Predicting complete response: is there a role for non-operative management of rectal cancer? J Gastrointest Oncol (2015) 6(2):241–6. doi: 10.3978/j.issn.2078-6891.2014.110 PMC4311100 25830042

[13] Habr-Gama A Perez RO Nadalin W Sabbaga J Ribeiro U Jr Silva E Sousa AH Jr. High 1-year complication rate after anterior resection for rectal cancer. J Gastrointest Surg (2014) 18(4):831–8. doi: 10.1007/s11605-013-2381-4 24249050

[14] Hiotis SP Weber SM Cohen AM Minsky BD Paty PB Guillem JG. Assessing the predictive value of clinical complete response to neoadjuvant therapy for rectal cancer:an analysis of 488 patients. J Am Coll Surg (2002) 194(2):131–5. doi: 10.1016/S1072-7515(01)01159-0 11848629

[15] Nancy YL Jiade JL. Target volume delineation and field setup: A practical guide for conformal and intensity-modulated radiation therapy. Berlin, Heidelberg: Springer-Verlag (2013) p. 161–8. doi: 10.1007/978-3-642-28860-9

[16] Weiser MR. AJCC 8th edition: Colorectal cancer. Ann Surg Oncol (2018) 25(6):1454–5. doi: 10.1245/s10434-018-6462-1 29616422

[17] Cao W Chen HD Yu YW Li N Chen WQ. Changing profiles of cancer burden worldwide and in China: A secondary analysis of the global cancer statistics 2020. Chin Med J (Engl) (2020) 134(7):783–91. doi: 10.1097/CM9.0000000000001474 PMC8104205 33734139

[18] Cheong C Shin JS Suh KW. Prognostic value of changes in serum carcinoembryonic antigen levels for preoperative chemoradiotherapy response in locally advanced rectal cancer. World J Gastroenterol (2020) 26(44):7022–35. doi: 10.3748/wjg.v26.i44.7022 PMC7701949 33311947

[19] Li A He K Guo D Liu C Wang D Mu X. Pretreatment blood biomarkers predict pathologic responses to neo-CRT in patients with locally advanced rectal cancer. Future Oncol (2019) 15(28):3233–42. doi: 10.2217/fon-2019-0389 31373223

[20] Das P Skibber JM Rodriguez-Bigas MA Feig BW Chang GJ Wolff RA. Predictors of tumor response and downstaging in patients who receive preoperative chemoradiation for rectal cancer. Cancer (2007) 109(9):1750–5. doi: 10.1002/cncr.22625 17387743

[21] Peng H Wang C Xiao W Lin X You K Dong J. Analysis of clinical characteristics to predict pathologic complete response for patients with locally advanced rectal cancer treated with neoadjuvant chemoradiotherapy. J Cancer (2018) 9(15):2687–92. doi: 10.7150/jca.25493 PMC6072814 30087709

[22] Motta R Ybazeta P Cordova O. Predictive factors of complete pathological response in operated patients with locally advanced rectal cancer after chemoradiotherapy neoadjuvant treatment in Peru. Ann Oncol (2018) 29(Suppl 5):v84. doi: 10.1093/annonc/mdy151.298

[23] Garland ML Vather R Bunkley N Pearse M Bissett IP. Clinical tumour size and nodal status predict pathologic complete response following neoadjuvant chem oradiotherapy for rectal cancer. Int J Colorectal Dis (2014) 29(3):301–7. doi: 10.1007/s00384-013-1821-7 24420737

[24] Wu F Wang J Yang C Zhou C Niu W Zhang J. Volumetric imaging parameters are significant for predicting the pathological complete response of pr eoperative concurrent chemoradiotherapy in local advanced rectal cancer. J Radiat Res (2019) 60(5):666–76. doi: 10.1093/jrr/rrz035 PMC6805984 31165155

[25] Garcia-Aguilar J Chow OS Smith DD Marcet JE Cataldo PA Varma MG. Effect of adding mFOLFOX6 after neoadjuvant chemoradiation in locally advanced rectal cancer: A multicentre, phase 2 trial. Lancet Oncol (2015) 16(8):957–66. doi: 10.1016/S1470-2045(15)00004-2 PMC4670237 26187751

[26] Liang HQ Dong ZY Liu ZJ Luo J Zeng Q Liao PY. Efficacy and safety of consolidation chemotherapy during the resting period in patients with local advanced rectal cancer. Oncol Lett (2019) 17(2):1655–63. doi: 10.3892/ol.2018.9804 PMC6341791 30675225

[27] Zhai Z Zhang K Wang C Zhang T Wang L Yao J. Adding three cycles of CAPOX after neoadjuvant chemoradiotherapy increases the rates of complete response for locally advanced rectal cancer. Curr Oncol (2021) 28(1):283–93. doi: 10.3390/curroncol28010033 PMC7903282 33419188

[28] Kim SY Joo J Kim TW Hong YS Kim JE Hwang IG. A randomized phase 2 trial of consolidation chemotherapy after preoperative chemoradiation therapy versus chemoradiation therapy alone for locally advanced rectal cancer: KCSG CO 14-03. Int J Radiat Oncol Biol Phys (2018) 101(4):889–99. doi: 10.1016/j.ijrobp.2018.04.013 29976501

[29] Moore J Price T Carruthers S Selva-Nayagam S Luck A Thomas M. Prospective randomized trial of neoadjuvant chemotherapy during the 'wait period' following preoperative chemoradiotherapy for rectal cancer: Results of the WAIT trial. Colorectal Dis (2017) 19(11):973–9. doi: 10.1111/codi.13724 28503826

[30] Riesco-Martinez MC Fernandez-Martos C Gravalos-Castro C Espinosa-Olarte P La Salvia A Robles-Diaz L. Impact of total neoadjuvant therapy vs. standard chemorad-iotherapy in locally advanced rectal cancer: A systematic review and meta- analysis of randomized trials. Cancers (Basel) (2020) 12(12):3655. doi: 10.3390/cancers12123655 33291454 PMC7762140

[31] Petrelli F Trevisan F Cabiddu M Sgroi G Bruschieri L Rausa E. Total neoadjuvant therapy in rectal smith NJ, shihab O, arnaout a, et al. MRI for detection of extramural vascular invasion in rectal cancer. AJR Am J Roentgenol (2008) 191(5):1517–22. doi: 10.1097/SLA.0000000000003471 18941094

[32] Smith NJ Shihab O Arnaout A Swift RI Brown G. MRI For detection of extramural vascular invasion in rectal cancer. AJR Am J Roentgenol (2008) 191(5):1517–22. doi: 10.2214/AJR.08.1298 18941094

[33] Smith NJ Barbachano Y Norman AR Swift RI Abulafi AM Brown G. Prognostic significance of magnetic resonance imaging-detected extramural vascular invasion in rectal cancer. Br J Surg (2008) 95(2):229–36. doi: 10.1002/bjs.5917 17932879

[34] van den Broek JJ van der Wolf FSW Heijnen LA Schreurs WH. The prognostic importance of MRI detected extramural vascular invasion (mrEMVI) in locally advanced rectal cancer. Int J Colorectal Dis (2020) 35(10):1849–54. doi: 10.1007/s00384-020-03632-9 32488420

[35] Chand M Bhangu A Wotherspoon A Stamp GWH Swift RI Chau I. EMVI-positive stage II rectal cancer has similar clinical outcomes as stage III disease following pre-operative chemoradiotherapy. Ann Oncol (2014) 25(4):858–63. doi: 10.1093/annonc/mdu029 24667718

[36] Prampolini F Taschini S Pecchi A Sani F Spallanzani A Gelsomino F. Magnetic resonance imaging performed before and after preoperative chemoradiotherapy in rectal cancer: predictive factors of recurrence and prognostic significance of MR-detected extramural venous invasion. Abdom Radiol (NY) (2020) 45(10):2941–9. doi: 10.1007/s00261-018-1838-z 30483843

[37] 2017 European Society of Coloproctology (ESCP) Collaborating Group. Evaluating the incidence of pathological complete response in current international rectal cancer practice: the barriers to widespread safe deferral of surgery. Colorectal Dis (2018) 20(Suppl 6):58–68. doi: 10.1111/codi.14361 30255641

[38] Hammarström K Imam I Mezheyeuski A Ekström J Sjöblom T Glimelius B. A comprehensive evaluation of associations between routinely collected staging information and the response to (Chemo)Radiotherapy in rectal cancer. Cancers (Basel) (2020) 13(1):16. doi: 10.3390/cancers13010016 33375133 PMC7792936

[39] Sun Y Li J Shen L Wang X Tong T Gu Y. Predictive value of MRI-detected extramural vascular invasion in stage T3 rectal cancer patients before neoadjuvant chemoradiation. Diagn Interv Radiol (2018) 24(3):128–34. doi: 10.5152/dir.2018.17286 PMC5951200 29770764

[40] Park CH Kim HC Cho YB Yun SH Lee WY Park YS. Predicting tumor response after preoperative chemoradiation using clinical parameters in rectal cancer. World J Gastroenterol (2011) 17(48):5310–6. doi: 10.3748/wjg.v17.i48.5310 PMC3247696 22219601

[41] Lee SY Kim CH Kim YJ Kwak HD Ju JK Kim HR. Obesity as an independent predictive factor for pathologic complete response after neoadjuvant chemor adiation in rectal cancer. Ann Surg Treat Res (2019) 96(3):116–22. doi: 10.4174/astr.2019.96.3.116 PMC6393413 30838183

[42] Russo AL Ryan DP Borger DR Wo JY Szymonifka J Liang WY. Mutational and clinical predictors of pathologic complete response in the treatment of locally advanced rectal cancer. J Gastrointest Cancer (2014) 45(1):34–9. doi: 10.1007/s12029-013-9546-y PMC4361942 24006244

[43] Appelt AL Pløen J Harling H Jensen FS Jensen LH Jørgensen JC. High-dose chemoradiotherapy and watchful waiting for distal rectal cancer: A prospective observational study. Lancet Oncol (2015) 16(8):919–27. doi: 10.1016/S1470-2045(15)00120-5 26156652

[44] Bertocchi E Barugola G Nicosia L Mazzola R Ricchetti F Dell'Abate P. A comparative analysis between radiation dose intensification and conventional fractionation in neoadjuvant locally advanced rectal cancer: A monocentric prospective observational study. Radiol Med (2020) 125(10):990–8. doi: 10.1007/s11547-020-01189-9 32277332

